# Antigenotoxic and Life-Prolonging Effects of Flavoured Kombuchas on *Drosophila melanogaster*

**DOI:** 10.17113/ftb.62.02.24.8308

**Published:** 2024-06

**Authors:** Ayşen Yağmur Burgazlı, Ghada Tagorti, Burçin Yalçın, Merve Güneş, Berfin Eroğlu, Eda Delik, Burcu Emine Tefon Öztürk, Bülent Kaya

**Affiliations:** Biology Department, Faculty of Science, Akdeniz University, Dumlupınar Boulevard, 07058, Antalya, Türkiye

**Keywords:** antigenotoxicity, *Drosophila melanogaster*, kombucha, lifespan

## Abstract

**Research background:**

Kombucha is a fermented beverage with several health benefits; however, to improve its antioxidant activity, new raw materials such as hop, madimak and hawthorn were included in the present study.

**Experimental approach:**

The somatic mutation and recombination test (SMART) was performed on the fruit fly (*Drosophila melanogaster*) to evaluate the antigenotoxic potential of black tea-flavoured kombucha and three other flavours of kombuchas (hop, madimak and hawthorn) against H_2_O_2_- and K_2_Cr_2_O_7_-induced genotoxicity. Furthermore, a lifespan assay was performed to assess the effects of kombuchas on the longevity of the fruit fly.

**Results and conclusions:**

According to the results obtained from the SMART assay, hop-flavoured kombucha attenuated genotoxicity induced by H_2_O_2_, and madimak-flavoured kombucha reduced genotoxicity induced by H_2_O_2_ and K_2_Cr_2_O_7_. Black tea- and hop-flavoured kombucha prolonged the lifespan of the fruit fly (*Drosophila melanogaster*) after the treatment with H_2_O_2_ and K_2_Cr_2_O_7_.

**Novelty and scientific contribution:**

Hop-flavoured kombucha is a promising antioxidant that protects the genome and extends the lifespan of the fruit fly. This study sheds light on novel beverages that can combat ageing and protect against genotoxicity.

## INTRODUCTION

Kombucha is a fermented beverage made by adding sugar to the infusion of black, green or oolong tea (*Camellia sinensis*) leaves and the inoculum called symbiotic culture of bacteria and yeast (SCOBY) (*1*). The consumption of kombucha began in China around 220 BC and in Japan around 414 AD due to its energising and detoxifying properties (*2*). Due to its functional properties, kombucha has become one of the most popular fermented beverages with low-alcohol content on the global market (*3*).

Several compounds including vitamins (*e.g.* B2, B6, B12 and vitamin C), minerals (*e.g.* Mn, Fe, Zn, Cu and Ni), acids (*e.g.* acetic, citric and gluconic acids) and polyphenols, especially catechins, have been identified in kombucha (*4*). However, the composition of the beverage differs depending on the raw materials and fermentation parameters such as time and speed (*5*, *6*). To enhance the antioxidant potential and antimicrobial activity of kombucha, alternative materials, such as grape juice (*7*), soy (*8*) and banana peel (*9*), have been used.

*Humulus lupulus* (hop) is a plant from the Cannabeaceae family, native to Europe, Lithuania, Asia and North America, which has been used in traditional medicine to treat anxiety, fever and gastric problems (*10*, *11*). Later, hop was used as a flavouring compound in the brewing industry (*12*). Hop is rich in flavonoids, including flavonols, flavanones and especially prenylflavonoids (*13*). Until recently, the antioxidant properties of hops were associated with the prenylflavonoids known as xanthohumol and its derivatives (isoxanthohumol) (*14*, *15*).

*Polygonum cognatum* (madimak) is a plant member of the Polygonaceae family, a species from Türkiye, that is generally used to treat gingivitis and enhance diuresis (*16*, *17*). Madimak exhibits antioxidant effects given the abundance of coumarin, quercetin, sinapic and salicylic acid (*16*, *18*).

*Crataegus monogyna* (hawthorn) is a plant from the Rosaceae family, which is native to Europe, northwest Africa and western Asia, and is often used in folk medicine for the treatment of diabetes and asthma. Hawthorn also has antioxidant properties due to its polyphenolic and flavonoid compounds (*19*-*21*).

In this study, the antigenotoxic potential of black tea-flavoured kombucha and kombucha with other three flavours (hop, madimak and hawthorn) against hydrogen peroxide and potassium dichromate(VI) was investigated, and their effects on the lifespan of a fruit fly (*Drosophila melanogaster*) were evaluated. *Drosophila* is a promising model organism for studying antigenotoxicity and longevity (*22*, *23*). *Drosophila melanogaster* has a short life cycle, a rapid reproductive rate and around 77 % of its genetic content encompasses orthologous disease-related genes in humans (*24*).

The wing SMART assay is an *in vivo* test to evaluate the genotoxicity and antigenotoxicity of several chemicals such as plant extracts, foods and drugs in the somatic cells of *Drosophila melanogaster* (*25*). Lifespan assay requires large populations and well-maintained animal stocks suitable for short-lived fruit flies (*26*).

## MATERIALS AND METHODS

### Chemicals

The black tea-flavoured kombucha and other three flavours of kombucha (hop, madimak and hawthorn) were obtained from Tefon Öztürk's Laboratory at Akdeniz University (Antalya, Türkiye). The composition of the tested kombuchas and the process of preparation were published in our previous study (*27*). Hydrogen peroxide and potassium dichromate(VI) were purchased from Sigma-Aldrich, Merck (St. Louis, MO, USA).

### Wing SMART test on Drosophila

Somatic mutation and recombination test (SMART) is an *in vivo* test frequently used to detect point mutations, deletions, non-disjunctions and recombinations (*28*). The principle of the SMART test on *Drosophila* is based on the loss of heterozygosity using two strains: *flare-3* (*flr^3^/ln* (*3LR*) *TM3, Bd^S^*) and multiple wing hair (*mwh/mwh*) (*29*, *30*).

Eggs were collected for eight hours from a standard cross between male (*mwh*) and female (*flr^3^*) flies. Once the collected eggs reached the larval stage, third-instar larvae ((72±4) hours old) were collected and subjected to chronic treatment in vials containing 4.5 g of *Drosophila* instant medium (Carolina Biological Supply Co., Burlington, NC, USA) soaked in 9 mL of the tested compounds. Distilled water was used as a negative control, whereas H_2_O_2_ (0.05 M) and K_2_Cr_2_O_7_ (1 mM) were used as positive controls. The flies were stored in 70 % ethanol at 4 °C until further use. The wings of the adult flies were embedded in Faure’s solution (50 g chloral hydrate, 30 g gum Arabic, 20 mL glycerol and 50 mL distilled water) and mounted on glass slides to evaluate the mutant spots under an optical microscope at 400× magnification. The mutant spots were classified as large single spots (≥2 cells of *mwh* type or ≥4 cells of *flr^3^* type), small single spots (1−2 cells of *mwh* type) and twin spots (*mwh* and *flr^3^* cells) (*28*). Eighty wings were counted in each treatment group. A chronic exposure (48 h) in three replicates was performed.

### The assay of Drosophila lifespan

Each vial contained 50 newly enclosed Oregon R+ strain of *Drosophila* treated with four different experimental food media (black tea, hop, madimak and hawthorn), with three replicates for each condition. Each type of food medium was mixed with toxic compounds, including 0.05 M H_2_O_2_ or 1 mM K_2_Cr_2_O_7_. Distilled water was used as a negative control. The food medium was renewed twice a week and dead flies were counted every 48 h.

### Statistical analysis

The SMART was performed using the multiple-decision procedure with the conditional binomial test (*31*, *32*). The probability level (α=β) was set at 0.05. Kaplan-Meier survival analysis was done and survival curves were analysed with the log-rank test. The mean value of the 10 % of flies that had the longest lifespan was considered as the maximum lifespan. The statistical significance of the mean value and the maximum lifespan was determined using a one-way ANOVA followed by Dunnett’s *t*-test. Cox proportional regression was used to calculate the hazard ratio. The statistical analysis was performed with SPSS Statistics v. 22.0 (*33*) and RStudio (v. 2022.07.0+548) (*34*).

## RESULTS AND DISCUSSION

**Fig. 1:**
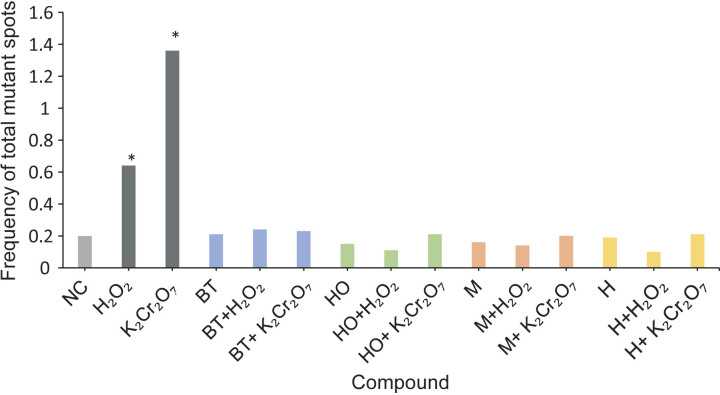
Influence of kombucha preparations on frequency of total mutant spots. NC=negative control, BT=black tea, HO=hop, M=madimak, H=hawthorn. *p<0.05 *vs* distilled water as negative control (conditional binominal test)

The kombucha preparations obtained after the fermentation process were used without further dilution and applied to the medium for *Drosophila*. Hydrogen peroxide is a reactive oxygen intermediate produced by physiological processes and/or exposure to xenobiotics. However, excessive production can lead to DNA damage, including single- and double-strand breaks and purine/pyrimidine oxidation (*35*). Potassium dichromate is a potent genotoxic agent causing chromosomal aberrations, oxidative stress, DNA breaks and lipid peroxidation (*36*). The frequencies of total mutant spots of H_2_O_2_ and K_2_Cr_2_O_7_ used in the present study were significantly higher than that of the negative control (distilled water) (Fig. 1), indicating that both H_2_O_2_ and K_2_Cr_2_O_7_ had mutagenic effects. This is consistent with the results of previous studies of *Drosophila* (*37*, *38*).

Both hop- and hawthorn-flavoured kombucha attenuated H_2_O_2_-induced genotoxicity, while madimak-flavoured kombucha reduced K_2_Cr_2_O_7_- and H_2_O_2_-induced genotoxicity by decreasing the frequency of total mutant spots compared to positive controls (Fig. 1). Based on our findings from a previous study (*27*), the tested madimak-flavoured kombucha has a higher chlorogenic acid concentration than hop and hawthorn kombuchas (868.4 *vs* 103 *vs* 15.5 mg/mL). Chlorogenic acid may exert antigenotoxic effects by reducing oxidative stress and maintaining enzymatic antioxidant activities (*39*, *40*). No significant change in the frequency of total mutant spots was reported for black tea-flavoured kombucha, which could be due to the absence of protocatechuic acid in its chemical composition compared to flavoured kombucha (*27*). As reported in a previous study, protocatechuic acid showed an antigenotoxic effect against H_2_O_2_ in the wing SMART assay of *Drosophila* (*41*). In addition, gallic acid, a well-known antioxidant, was the major compound in hop- and hawthorn-flavoured kombuchas (*27*).

The effects of various combinations of kombucha (black tea, hop, madimak and hawthorn) alone or supplemented with potentially toxic agents (H_2_O_2_ or K_2_Cr_2_O_7_) on the lifespan of the Oregon R+ strain of *Drosophila* were evaluated.

**Fig. 2:**
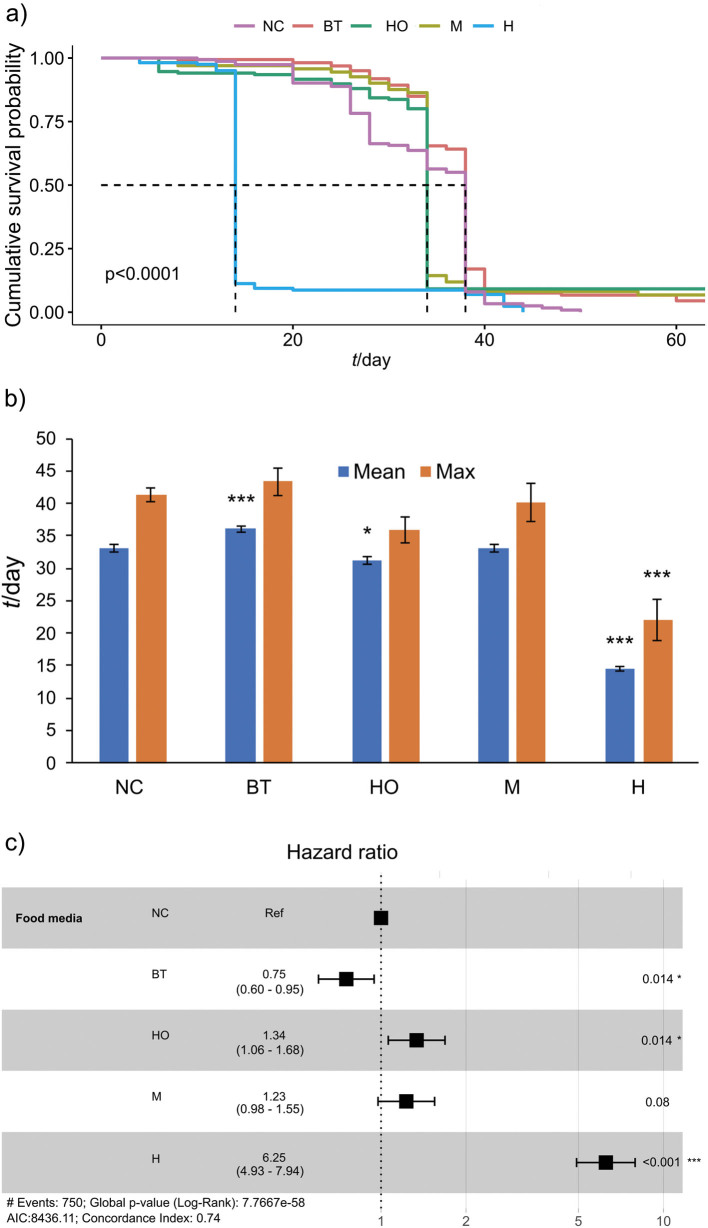
Effect of kombucha (black tea, hop, madimak and hawthorn) alone on the lifespan parameters in Oregon R+ strain of flies: a) survival curves, b) mean and maximum lifespan±standard error, and c) hazard ratios and 95 % confidence intervals. Asterisks indicate the level of statistical significance (*p<0.05, ***p<0.001, log-rank test (a), Dunnett’s *t*-test compared with NC (b), Cox proportional hazards regression (c)). *N*(fly)=150 for each condition. NC=negative control (distilled water), BT=black tea, HO=hop, M=madimak, H=hawthorn

According to Fig. 2a, black tea, hop and madimak kombucha increased the cumulative survival probability compared to the negative control (distilled water) (log-rank, χ^2^=440, p<0.0001). However, only the mean value for black tea-flavoured kombucha (36.04 days) statistically significantly increased (8.6 %) compared to the negative control (Fig. 2b). In addition, the hazard ratio was calculated to obtain information about the relative likelihood of the mortality of *Drosophila* after the exposure to different compounds. Interestingly, the black tea-flavoured kombucha had a promising potential to reduce the mortality by 25 % compared to the negative control (hazard ratio of 0.75) (Fig. 2c).

**Fig. 3:**
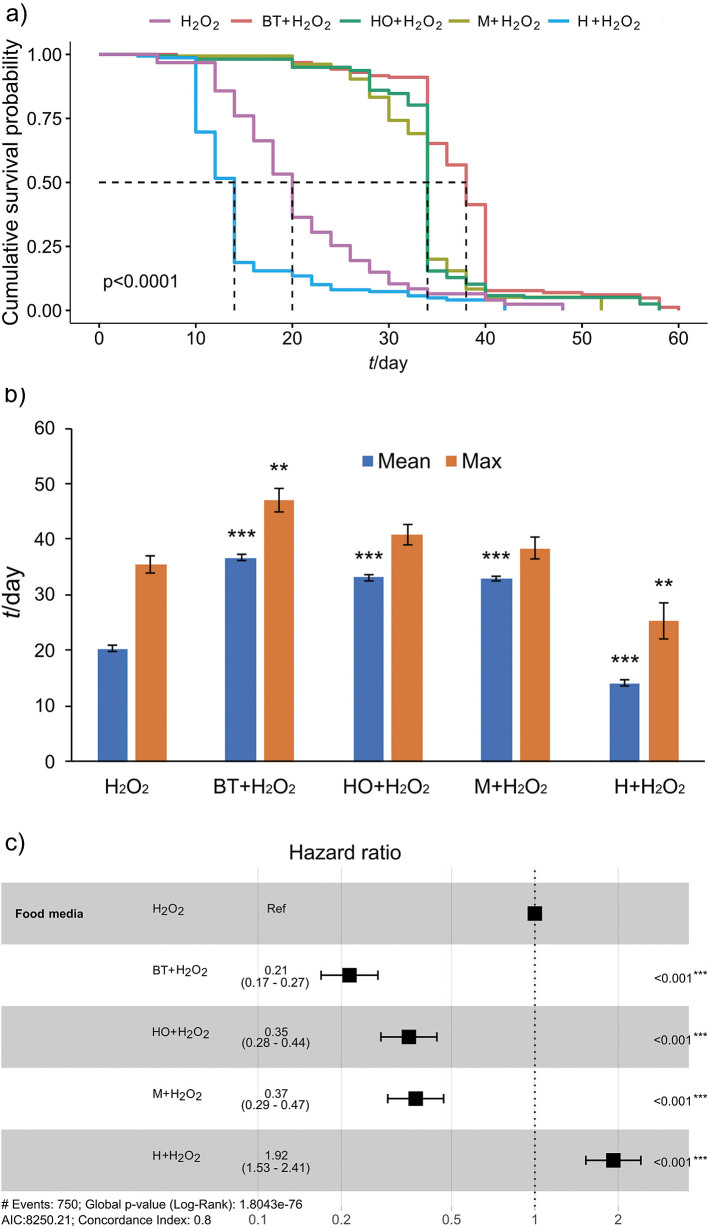
Effect of kombucha (black tea, hop, madimak and hawthorn) supplemented with hydrogen peroxide (0.05 M) on lifespan parameters in Oregon R+ strain of flies: a) survival curves, b) mean and maximum lifespan±standard error, and c) hazard ratios and 95 % confidence intervals. Asterisks indicate the level of statistical significance (**p<0.01, ***p<0.001, log-rank test (a), Dunnett’s *t*-test compared to H_2_O_2_ (b), Cox proportional hazards regression (c)). *N*(fly)=150 for each condition. BT=black tea, HO=hop, M=madimak, H=hawthorn

The greatest increase in cumulative survival probability was observed with black tea-flavoured kombucha supplemented with H_2_O_2_ (log-rank, χ^2^=592, p<0.0001), followed by hop kombucha with H_2_O_2_ and madimak kombucha with H_2_O_2_ compared to the treatment with H_2_O_2_ alone (Fig. 3a). In addition, the mean lifespan of *Drosophila* supplemented with black tea-flavoured kombucha (36.7 days) increased by 81 % and the maximum lifespan (47.1 days) increased by 32.2 % compared to H_2_O_2_ alone (Fig. 3b). The mean lifespan of flies supplemented with hop kombucha (33.1 days) increased by 63.1 % and the mean lifespan of flies supplemented with madimak kombucha (32.9 days) increased by 61.9 % compared to H_2_O_2_ alone (p<0.001). The hazard ratio of 0.21, 0.35 and 0.37 for black tea with H_2_O_2_, hop with H_2_O_2_, and madimak with H_2_O_2_, respectively, compared to H_2_O_2_ suggested that these combinations reduced the risk of mortality by 79, 65 and 63 %, respectively (Fig. 3c).

**Fig. 4:**
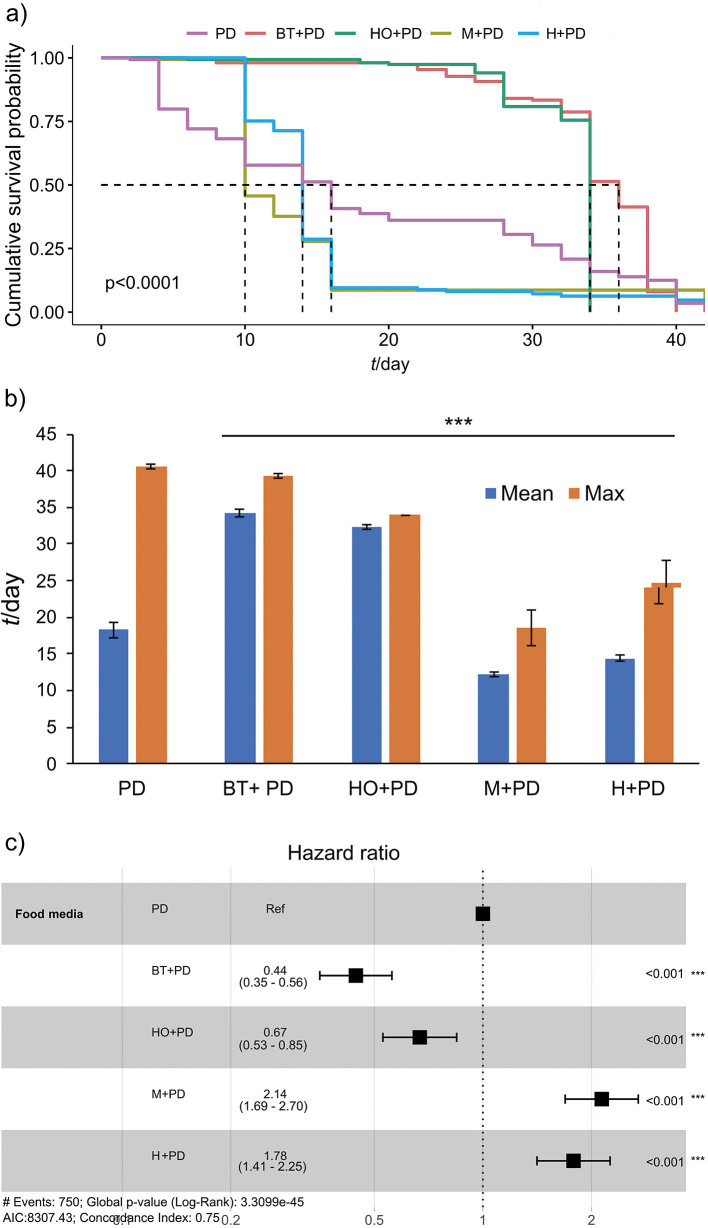
Effect of kombucha (black tea, hop, madimak and hawthorn) supplemented with potassium dichromate (1 mM) on the lifespan parameters in Oregon R+ strain of flies: a) survival curves, b) mean and maximum lifespan±standard error, and c) hazard ratios and 95 % confidence intervals. Asterisks indicate the level of statistical significance (***p<0.001, log-rank test (a), Dunnett’s *t*-test compared to PD (b), Cox proportional hazards regression (c)). *N*(fly)=150 for each condition. PD=potassium dichromate(VI), BT=black tea, HO=hop, M=madimak, H=hawthorn

The lifespan of flies that consumed different combinations of kombucha with K_2_Cr_2_O_7_ did not increase compared to K_2_Cr_2_O_7_ alone (log-rank, χ^2^=285, p<0.0001) (Fig. 4a). The median lifespan of flies supplemented with black tea-flavoured kombucha with K_2_Cr_2_O_7_ (36 days) increased by 125 %, the mean lifespan (34.2 days) increased by 86.9 %, while the maximum lifespan decreased by 3 % compared to the treatment with K_2_Cr_2_O_7_ alone (Fig. 4a and Fig. 4b). The median lifespan of hop kombucha with K_2_Cr_2_O_7_ (34 days) increased by 112.5 %, the mean lifespan (32.3 days) increased by 76.7 %, while the maximum lifespan decreased 16.1 % compared to K_2_Cr_2_O_7_ alone. With both madimak- and hawthorn-flavoured kombucha with K_2_Cr_2_O_7_, the median, mean and maximum lifespan decreased. As shown in Fig. 4c, madimak- and hawthorn-flavoured kombucha with K_2_Cr_2_O_7_ had higher hazard ratio than K_2_Cr_2_O_7_ alone and increased the risk of mortality by 114 and 78 %, respectively.

There is limited data on the effect of kombucha on the lifespan of the fruit fly. To the best of our knowledge, no kombucha preparations have been used for fruit flies and no study has been conducted on kombucha preparations with *Humulus lupulus* (hop), *Crataegus monogyna* (hawthorn) and *Polygonum cognatum* (madimak). The concentration and the type of phenolic compounds can interfere with the bioactive effects of kombucha. According to our previous study (*23*), the tested black tea-flavoured kombucha (14 days of fermentation) had the highest antioxidant activity (655 µmol/mL), followed by hop-flavoured kombucha (634 µmol/mL). In addition, a strong correlation was found between the total flavonoid content and the antioxidant activity (r=0.69, p<0.05). The antioxidant activity of black tea-flavoured kombucha could be related to the processes that tea (*Camellia sinensis* L.) leaves undergo during preparation, which activate polyphenol oxidases, leading to catechin oxidation and the production of theaflavins and thearubigins (*42*). Various studies have been conducted on some ingredients that are consistent with our study. Black tea extract (commercial brand without further dilution) has been reported to increase the median lifespan of Oregon R strain of *Drosophila* by 20.2 % (*43*).

Hops are rich in xanthohumol, which was later reported to increase the median lifespan by 10.41 % and the maximum lifespan by 8.88 % in Oregon R-C strain of *Drosophila* at a concentration of 0.5 mg/mL (*44*). It is worth noting that in some cases a single component can have a negative effect. For example, caffeine at a concentration of 0.03 mg/mL reduced median lifespan by 22.86 % and the maximum lifespan by 11.6 % in Canton-S strain of *Drosophila* (*45*). However, black tea-flavoured kombucha, which contains the highest amount of caffeic acid as a mixture of polyphenols, extended the lifespan of the fruit fly.

## CONCLUSIONS

Kombucha is a fermented beverage with a high content of probiotics. To enhance the beneficial properties of this drink, studies are being conducted on kombucha flavoured with beneficial herbs. This is the first study to investigate the effects of kombucha drinks flavoured with hawthorn, hop and madimak on the lifespan of the fruit fly (*Drosophila melanogaster*). Hop-flavoured kombucha has an antigenotoxic effect and can extend the lifespan of *Drosophila* treated with H_2_O_2_ and K_2_Cr_2_O_7_, although its antioxidant composition is similar to that of black tea-flavoured kombucha and the other flavoured kombuchas. However, further studies are needed to evaluate the remaining genotoxicity endpoints, such as DNA breaks, to ensure the safety of hop-flavoured kombucha for consumers.
